# Institutional Questions

**Published:** 1899-09-23

**Authors:** 


### INSTITUTIONAL QUESTIONS.

### A Delicate Point.

"A Correspondent" asks for advice on the following question: A small hospital has to rent a neighbouring house as additional accommodation (principally for the nursing staff), containing more rooms than are actually required for its needs. Is there any reason why the Committee of Management should not allow these extra rooms to be retained for the use of the medical officer for private patients, these paying the hospital so much per week?

There cannot be any reason why, if a hospital has more beds than it requires, these should not be made use of by the management for the reception of private patients, it being understood that such patients' payments fully cover cost of board and lodging and nursing, and that they pay their own medical attendant. We do not think, however, that these beds should be exclusively reserved for the use of any one medical man, or that the medical officer attached to any public institution should make use of his connection therewith to secure such an exclusive privilege for himself.

### Polyclinics.

"Hospital Worker" writes: (1) Where can I obtain information concerning the management of what may be termed "Polyclinics"? I mean a scheme whereby the out-patient departments of the various hospitals and infirmaries in a town may be brought under one roof and administered by a joint board. Is such a scheme in operation anywhere? The Vienna Polyclinic is quite a different institution to that indicated by its name. (2) Also where shall I find information respecting the treatment of inebriates, and the institutions existing in this country for their reception?

(1) We are not able to find that a scheme for centralising the out-patient departments in a large town has been adopted anywhere. In principle the plan is most nearly carried out in Paris, where the Assistance Publique is, we believe, administered from a central bureau, patients applying for indoor treatment being thence allotted to the various hospitals under the management of this body. There would doubtless be advantages attached to such a course if pursued in connection with out-patients. It would be economical, would save the patients time and worry, and probably ensure their obtaining the special treatment fitted for their case with greater certainty than under the present system. It would also do away with what may be called the competition for patients between the various hospitals, for under this scheme admission as in-patients to the various hospitals would, except in the case of accidents or emergencies, probably be reserved exclusively for cases sent on from the central office. In carrying such a scheme into practice, however, there would be many almost insuperable difficulties, not the least of which would be the minimising of the personal interest now taken by individual subscribers in particular hospitals, which would surely lead to a decrease in subscriptions and bequests. Its establishment would be a step in the direction of the municipalisation of hospitals, a result sincerely to be deprecated by their best friends. (2) With regard to the second question, the treatment of alcoholics, we may refer you to "Helps in Sickness and to Health," by Sir Henry Burdett, K.C.B., published by the Scientific Press, where you will find a short chapter devoted to this subject, with a list of licensed and unlicensed retreats and homes for inebriates.

**Cottage Hospital Management.**

"A Member of Committee" writes: We have a small cottage hospital in our neighbourhood, containing six beds. At present we have only one resident nurse in charge, extra trained assistance being obtained from the nearest town when there are bad cases. But the precise moment when the work becomes more than enough for one nurse to manage properly is not always easy to fix, and it seems to some members of the Committee of Management that this arrangement taxes too severely our charge nurse, who with only one servant has a great deal of responsibility connected with housekeeping and other matters, in addition to the actual nursing of her patients. Please give me your opinion on this point.

Certainly, when your beds are full, the sole charge is more than one nurse can properly be asked to undertake, without putting too great a strain upon her. Even with two or three patients a conscientious nurse, single-handed, will find it very difficult to secure for herself such an amount of sleep and off-duty time as will enable her to continue her work for long without injuring her health. One bad case is sufficient to keep her tied to the house for days at a time, and even if nurses are willing, as we know they are, to forego both rest and needful exercise and fresh air when the "wards are heavy," those who are responsible for their employment should be most scrupulous to see that no such undue sacrifice is exacted from them. Moreover, in a small hospital where the charge nurse is responsible for the housekeeping and management expenses, and is being constantly urged to be economical, she will hesitate too often to ask for the "extra help" which will add in no small degree to the outlay at the end of the year. A common course to take under such circumstances is to have a "paying probationer," who can, under the training of a competent nurse and the medical officer, soon become a fairly efficient assistant, and capable of taking charge of the patients in the absence of the nurse. There is usually little difficulty in finding well-educated girls, not old enough to enter upon training in a general hospital, who are glad to gain preliminary experience in a small institution, and to pay for their board and lodging, but it would seem a better plan, if possible, to obtain as a servant someone who has had enough nursing experience to enable her at times to take temporary charge in the wards. There must be many nurses who have been driven by circumstances to take up "service," and a person of this sort would probably be found useful as a servant in such an institution as the above. The engagement of a "paying probationer" would no doubt be very helpful to the cottage hospital, but we hesitate to recommend a young woman to undertake work which could only give her such an imperfect training as such a cottage hospital could provide.

**Hospital Soup Kitchens.**

"E. B." asks: Can you tell me if the plan, which I believe is sometimes adopted, of having a soup kitchen in connection with a village hospital, is found to work well in practice, and if it is one you would recommend?

The idea of working a soup kitchen from a hospital is not often put into practice, so far as we know, but if properly managed there seems no reason why it should not answer well and be a very real boon to the sick poor in any district. At the Acton Cottage Hospital, built by Mr. Passmore Edwards not very long ago, such a plan is in operation, and we believe is found to be quite satisfactory. The food is given out at stated times on the presentation of tickets, which have, we understand, to be signed by the medical man. It is certain that amongst the poor more can be done by feeding than by medicine in many cases, and the provision of nourishment in this way may be looked upon properly as part of medical treatment. On the whole, always provided that such a scheme does not degenerate into a pauperising agency, the establishment of a hospital kitchen can be safely recommended.

